# Optimal place of treatment for young infants aged less than two months with any low-mortality-risk sign of possible serious bacterial infection: Study Protocol for a randomised controlled trial from low- and middle-income countries

**DOI:** 10.7189/jogh.13.04055

**Published:** 2023-07-14

**Authors:** PSBI Study Group, Abdullah H Baqui,, Rasheda Khanam, Mohammod Shahidullah, Salahuddin Ahmed, Arunangshu Dutta Roy,, Iffat Ara Jaben,, Manajjir Ali, Muhammad Shariful Islam, Sabina Ashrafee Lipi, Md Jahurul Islam, Amha Mekasha, Abiy Seifu, Lulu Muhe, Damen Hailemariam, Bogale Worku, Solome Jebessa, Temsunaro Rongsen-Chandola, Nidhi Goyal, Amit Kumar, Nita Bhandari, Shayam Kaushik, Surjeet Kumar, Amitabh Jain, Mangla Sood, Rakesh Sharma, Jagjit Singh Dalal, Kundan Mittal, GP Kaushal, Vineeta Wadhwa, Vishwajeet Kumar, Aarti Kumar, Rashmi Kumar,, Vinay Pratap Singh, Pramod Kumar Singh, Vivek Kumar Singh, Yashwant Kumar Rao, Krishna Kumar Dokania, Ved Prakash, Shiv Kumar, Robinson Daniel Wammanda, Laila Hassan, Saraja Ahmodu Opaluwa, Ishaku Hassan, Aminu Shadrach Adamu, Bawa Ega, Benazir Baloch, Imran Nisar, Fyezah Jehan, Karim Manji, Christopher Robert Sudfeld, Rodrick Kisenge, Nahya Salim, Sarah Somji, Mohamed Kheri Bakari, George Kibogoyo, Kristina Lugangira, Veneranda M Ndensangia, Christopher Paul Duggan, Rajiv Bahl, Karen Edmond, Sachiyo Yoshida, Shamim A Qazi, Yasir Bin Nisar

## Abstract

**Background:**

World Health Organization (WHO) recommends hospitalisation and injectable antibiotics for clinical sepsis / possible serious bacterial infection (PSBI) in young infants up to two months of age. However, some young infants with low-mortality risk signs of PSBI may not require hospitalisation, for which evidence needs to be generated.

**Methods:**

This is a protocol for a multicentre, individually randomised, open-label trial that will be conducted in seven sites in six countries Bangladesh, Ethiopia, India (two sites), Nigeria, Pakistan and Tanzania. All sites will use this common protocol with the same study design, inclusion of participants, intervention, comparison, and outcomes, as well as quality control and analysis procedures to contribute to the overall sample size. All young infants (age <60 days) presenting at study hospitals with any single low-mortality risk sign (high body temperature ≥38°C, severe chest indrawing, or fast breathing of ≥60 breaths per minute in <7 days old infants) will be randomised to either outpatient care with injectable gentamicin for two days and oral amoxicillin for seven days (intervention) or inpatient care with injection gentamicin plus injection ampicillin along with supportive treatment, where needed, for seven days (control). We plan to enrol 7000 eligible young infants, 3500 infants in each of the two study arms. A trained and standardised independent outcome assessor will visit all enrolled cases on days two, four, eight and 15 post-randomisation to assess the study outcomes in both intervention and control groups. The primary outcome of poor clinical outcome, defined as death within two weeks of initiation of treatment, deterioration during the 7-day treatment period, or persistence of the presenting sign at the end of the 7-day treatment period, will be compared to assess if the outpatient treatment leads to superior or at least non-inferior clinical outcome than inpatient treatment. The selected sites have extensive research experience. The methods and all study procedures will be harmonised through central training of research staff by WHO, standardisation exercises for clinical signs, central data coordination centre and internal and external monitoring. Continuous evaluation of the enrolment by the sites will be carried out through regular calls, databased monitoring, and site visits by WHO monitors. This trial has received ethical approvals from the WHO and local site institutional ethics committees.

**Discussion:**

If the results show that young infants with any single low-mortality risk PSBI sign can be effectively and safely treated on an outpatient basis, it may substantially increase access to treatment for infants and families with poor access to health facilities. It may also reduce the human, financial and material costs to the health system and allow the currently overloaded health facilities to focus on more critically ill infants. This evidence will contribute toward making a case for reviewing the current WHO PSBI management guideline.

**Registration:**

International Standard Randomised Controlled Trial Number ISRCTN44033252.

Neonatal mortality has substantially reduced over the last few decades, but an estimated 2.4 million neonatal deaths still occur worldwide annually, accounting for 47% of under-five deaths [1]. Neonatal infections account for over 35% of all neonatal deaths in South Asia and sub-Saharan Africa [2]. The World Health Organization (WHO) Integrated Management of Childhood Illness (IMCI) algorithm classifies neonates and young infants with clinically suspected sepsis as “Possible Serious Bacterial Infection (PSBI)” [3]. This classification is based on seven clinical signs – fast breathing (≥60 breaths per minute) in 0-6 days old babies, severe chest indrawing, high body temperature (≥38°C), low body temperature (<35.5°C), not able to feed at all or not feeding well / stopped feeding well, convulsions, and movement only when stimulated or no movement at all [3]. WHO guideline recommends that young infants with any sign of PSBI should be managed in a hospital with injectable antibiotics and supportive care [4]. When referral to a hospital is not feasible, the WHO guideline recommends further classification of these infants into those who are critically ill (**Box 1**) and those who have a clinical severe infection (CSI) [5]. CSI can be managed on an outpatient basis with injectable gentamicin for two or seven days and oral amoxicillin for seven days based on clinical trials from Africa [6,7] and Asia [8,9].

Box 1 **Sub-classification of children with signs of possible serious bacterial infection when a referral is not feasible [5].**

Critical illness

• convulsions

• not able to feed at all

• no movement at all

Clinical Severe Infection

*Low-mortality risk signs*

• high body temperature (≥38°C*)

• severe chest indrawing

• fast breathing of ≥60 breaths per minute in <7 days old infants

*Moderate-mortality risk signs*

• low body temperature (<35.5°C*)

• movement only when stimulated

• not feeding well / stopped feeding well

*Thresholds based on axillary temperature.

Several countries have now adopted the WHO PSBI management guideline [5]. Implementation research in several countries in Africa and Asia on the WHO PSBI guideline has demonstrated that this guideline can be scaled up and that outpatient treatment is safe and effective when hospitalisation is not feasible [10-22]. It is estimated that 8%-12% of all young infants had at least one episode of PSBI within the first two months of life. Overall, approximately a quarter to half of the sick young infants in different settings accept hospital referrals [6,8,23]. However, hospitalisation also has risks, particularly that of nosocomial infections, including those from multi-drug resistant pathogens [24-27]. Therefore, only those young infants with signs of PSBI who have a favourable benefit-risk ratio should be hospitalised. Secondary observational analyses of AFRIcan NEonatal Sepsis Trial (AFRINEST) [6,7] data showed that some of the more common clinical signs of PSBI are associated with relatively low mortality [28]. Specifically, having only fever (temperature ≥38°C) in infants 0-59 days of age, only severe chest indrawing in infants 0-59 days of age, or only fast breathing in infants 0-6 days of age had relatively low case fatality risk (CFR) of 0.8%, 0.9% and 2.0%, respectively [28] as compared to movement only on stimulation (CFR = 4%) or low body temperature (CFR = 11.0%) which had higher mortality. Infants presenting with multiple signs of CSI (two or more) also had a moderate risk of mortality (CFR = 5.7%). As expected, signs of critical illness were associated with a very high risk of death (convulsions, CFR = 11.3%, unable to feed at all, CFR = 22.9% and no movements at all, CFR = 25.0%).

An important implication of these findings is that young infants with signs associated with a relatively lower risk of mortality (fast breathing with ≥60 breaths per minute in 0-6 days of age, or temperature ≥38°C or severe chest indrawing in 0-59 days of age) may not require a referral for inpatient treatment in a hospital for one week per current WHO guidelines. If infants with low-risk signs of PSBI could be managed at the outpatient level, it may reduce the referral for hospitalisation by over 70%. On the other hand, infants who have the other signs of CSI (stop feeding well, movements only on stimulation, low body temperature), with multiple signs of CSI, as well as those who have a critical illness, are at a high risk of mortality [28] and may have a more favourable benefit-risk ratio for hospitalisation.

When mortality data from AFRINEST were analysed by place of treatment for all infants classified as PSBI, CFR was higher in hospitalised young infants as compared to infants treated on an outpatient basis when they refused referral for the same signs of CSI [28]. The overall CFR for young infants with CSI treated at the hospital was three times higher (6.5%) compared to those treated on an outpatient basis (1.9%) [28]. Nevertheless, due to the observational nature of the AFRINEST study and specifically that infants who refuse referral may be systematically different from those who accept, the findings cannot be considered causal. Therefore, there was a need to evaluate this in a randomised controlled trial.

The primary objective of this trial protocol is to measure the effect of outpatient treatment on poor clinical outcomes compared with inpatient treatment in young infants 0-59 days old with only one low-mortality risk sign of CSI. We hypothesise that the majority of infants with a single low mortality risk sign of CSI do not benefit from hospitalisation.

## METHODS

### Research question

Among young infants, 0-59 days of age with only one low-mortality risk CSI sign presenting to the outpatient / emergency department of a hospital (population), does outpatient treatment with injectable gentamicin for two days and oral amoxicillin for seven days (intervention), compared to the currently recommended inpatient hospital treatment initiated with injectable ampicillin and gentamicin and supportive care for seven days (control), result in lower rates of poor clinical outcome (death within two weeks of initiation of treatment, deterioration during the 7-day treatment period, or persistence of the presenting sign of CSI at the end of the 7-day treatment period) (outcome)?

### Study design, setting and sites

This will be a multicentre, individually randomised, controlled, open-label, two-arm trial. The study will be conducted in seven sites in six countries Bangladesh, Ethiopia, India (two sites), Nigeria, Pakistan and Tanzania (**Table 1**).

**Table 1:** Details of the study sites*

Study site	Description
Bangladesh	The study will be conducted in four hospitals. Zakaiganj Upazila Health Complex (ZUHC), Zakiganj (primary / public), Sunamganj District Hospital (SDH), Sunamganj (secondary / public), Moulvibazar District Hospital (MDH), Moulovibazar (secondary / public), and Habiganj District Hospital (HDH), Habiganj (secondary / public) in Sylhet. ZUHC has six paediatric beds and one radiant warmer, SDH has 14 beds, one incubator and three warmers, MDH has 17 beds and 17 warmers, HDH has 11 beds and 11 warmers in their Neonatal Intensive Care Units (NICU) / Special Neonatal Care Units (SNCU) respectively. All are functional 24 h a day. Except for ZUHC, all hospitals have trained paediatricians, but none have a neonatologist. Oxygen is available 24 h a day through cylinders.
Ethiopia	The study will be conducted in five hospitals. One named Tirunesh Beijing Hospital (secondary / public) in Addis Ababa, and four in the Oromia region named Bishoftu Hospital (secondary / public), Adama Hospital (secondary / public), Batu Hospital (General Hospital), Asella Hospital, Asella (Referral Hospital). Tirunesh Beijing Hospital has 22 beds and 17 incubators / warmers, Bishoftu Hospital has 15 beds and 17 incubators / warmers, Adama Hospital has 53 beds and 11 incubators / warmers, Batu Hospital has 18 beds and two incubators / warmers and Asella Hospital 40 beds and eight incubators / warmers in their NICU/SNCU respectively. All are functional 24 h a day. All hospitals have trained paediatricians, but none have a neonatologist. Oxygen is available 24 h a day through cylinders.
Himachal Pradesh-NCR, India	The study will be conducted in five hospitals ie, Dr YS Parmar Government Medical College Hospital (YSPGMC), Nahan (tertiary / public), Civil Hospital (CH), Poanta (secondary / public), Indira Gandhi Medical College Hospital (IGMC), Shimla (tertiary / Public), Pt BD Sharma Post Graduate Institute of Medical Sciences (PGIMS), Rohtak (tertiary / public), Dr Baba Saheb Ambedkar Hospital (BSAH), Delhi (tertiary / public). YSPGMC has 25 beds and six incubators / warmers, CH has eight beds and eight warmers, IGMC has 24 beds and 10 incubators / warmers, PGIMS has 65 beds, and 38 incubators / warmers and BSAH has 42 beds and 34 incubators/warmers in their NICU / SNCUs respectively, and all are functional 24 h a day. All hospitals have trained paediatricians, and only YSPGMC Nahan and PGIMS Rohtak hospitals have a neonatologist. Oxygen is available 24 h a day through central supply.
Uttar Pradesh, India	The study will be conducted in three hospitals ie, Hallet Hospital Kanpur (tertiary / public), Dufferin Hospital, Kanpur (secondary / public) and Shyam Children’s Charitable Hospital (SCCH), Kanpur (secondary / private). Hallet Hospital has 38 SNCU beds equipped with radiant warmers and 80 paediatric beds, Dufferin Hospital has 12 SNCU beds equipped with radiant warmers, and SCCH has 9 SNCU beds equipped with radiant warmers and 25 paediatric beds, and all are functional 24 h a day. All hospitals have trained paediatricians, and none has a neonatologist. Oxygen is available 24 h a day through central supply in Hallet Hospital and SCCH and cylinders in Dufferin Hospital.
Nigeria	The study will be carried out in three secondary / public hospitals ie, Hajiya Gambo Sawaba General Hospital (HSGH), Zaria, Giwa General Hospital (GGH), Giwa and Yusuf Dantsoho Memorial Hospital (YDMH), Tudun Wada, Kaduna. HSGH has 14 beds and one warmer (one incubator is on order), GGH has 11 beds and one warmer whereas YDMH has 15 incubators, 11 beds and 14 warmers in their NICUs / SNCUs respectively. All are functional 24 h a day. All hospitals are staffed by general-duty physicians. In addition, YDMH has a trained paediatrician / neonatologist. Oxygen is available 24 h a day either through central supply, oxygen concentrators and cylinders at the Hajiya Gambo Sawaba General Hospital and Yusuf Dantsoho Memorial Hospital while only through cylinders at the Giwa General Hospital.
Pakistan	The study will be done in two secondary-level hospitals, ie, The Aga Khan Hospital for Women and Children Hospital, Kharadar (AKU-KH), Karachi and Sindh Government Children Hospital (SGCH), and two tertiary care hospitals, ie, National Institute of Child Health (NICH), and Sind Institute of Child Health (SICH), Karachi. AKU-KH has five cots, four incubators, and three radiant warmers. SGCH has 16 incubators, 8 cots, 8 beds in Paediatric ICU, and 110 beds in the paediatric ward. NICH has 67 incubators, 27 cots, 330 beds in the Paediatric ward, 55 beds in the emergency and 24 functional ventilators. SICH has 65 incubators, 20 ward beds and 14 ICUs with 21 ventilators. Central oxygen 24 h a day and a radiant warmer are available. All four hospitals are functional with a facility of 24-h emergency service. Qualified paediatricians and one neonatologist each staff all hospitals.
Tanzania	The study will be carried out in two public / secondary / regional referral hospitals ie, Amana Hospital and Temeke Hospital. Amana Hospital has 59 cots and nine radiant warmers, whereas Temeke Hospital has 34 cots and seven radiant warmers in their NICUs / SNCUs, respectively. Both are functional 24 h a day. Both hospitals are staffed by qualified paediatricians, and none has a neonatologist. Oxygen is available 24 h a day through cylinders and oxygen concentrators.

*Additional hospitals may be included to increase enrolment depending upon the site-specific recruitment.

### Participants

All young infants 0-59 days old, presenting to outpatient clinics or emergency departments of participating hospitals, and living in a geographic area where follow-up for 14 days can be accomplished will be considered for inclusion in this study if they have only one of the following low-risk signs of PSBI: 1) body temperature ≥38°C, or 2) severe chest indrawing, or 3) fast breathing (≥60 breaths per minute) in <7 days old infants.

Infants will be excluded if they have any of the following: 1) weight <2 kg at the time of presentation (if age at screening is less than 10 days) or weight for age<-3z, or 2) signs of critical illness (no movement at all, unable to feed at all, convulsions), or 3) signs of CSI associated with a moderate risk of mortality (stopped feeding well, movement only on stimulation, low body temperature <35.5°C or two or more of the six signs of CSI), or 4) any sign suggestive of another serious illness/condition, such as major congenital malformations, severe jaundice, conditions requiring major surgery, meningitis, bone or joint infection, severe dehydration, hypoxaemia, etc., or 5) the appearance of low-mortality risk signs in the first 24 hours of life, or 6) hospitalised for any illness in the previous two weeks, or 7) prior use of injectable antibiotics in the last two days for the same illness except for pre-referral administration, or 8) previously included in this study or currently included in any other study.

### Intervention

Outpatient treatment with injectable gentamicin (once daily) for two days plus oral amoxicillin (twice daily) for seven days.

### Control

Inpatient antibiotic treatment initiated with WHO recommended antibiotic regimen – injectable ampicillin (twice or thrice a day, depending on age) plus injectable gentamicin (once daily) along with other supportive care for 7-10 days.

One of the possible reasons that outpatient care was observed to be better than hospital care in previous studies might be the poor quality of care in hospitals. In this study, we would like to reduce this factor as far as practically feasible. This means that the quality of care at the study hospitals will be reviewed against the WHO pocketbook for hospital care for children [4] and will be improved using quality improvement approaches to ensure a “minimum” quality of hospital care. Efforts to improve the quality of care will also be made at outpatient facilities.

### Screening and enrolment

Young infants up to two months of age in the outpatient or emergency department will be examined, triaged and stabilised by the consulting paediatrician or physician of the participating hospital. This process followed as part of regular facility procedures is referred to as ‘pre-screening’. If the paediatrician / physician observes signs of CSI (**Box 1**) during their initial assessment and the clinical condition of the infant is stable, the infant will be assessed by the study screening, enrolment and randomisation team (**Box 2**). After the consent has been obtained for screening, a study nurse / physician will screen the sick young infants in the outpatient or emergency department of the participating hospital, and information will be captured on the case report form (screening and enrolment form). Those who fulfil the above-mentioned inclusion criteria and do not have any exclusion criteria will be enrolled after confirmation of clinical signs by the treating physician after obtaining consent from the parents or caregivers. The study nurse / physician will fill out the appropriate case report form at this stage. Infants who are not eligible for enrolment will be managed as per the treatment protocols of the hospital.

Box 2 **Responsibilities of study teams.**

Screening, enrolment and randomisation team

• obtain consent for screening and perform screening of all young infants presenting at the outpatient or emergency department according to inclusion and exclusion criteria.

• communicate with the hospital nurse / physician to validate clinical findings.

• fill screening case report form.

• for enrolment in the trial communicate with the hospital nurse / physician, obtain consent for enrolment and fill the required case report form, complete randomisation process and assign treatment arm as inpatient and outpatient, share this information with study coordinators.

• share information about enrolled and non-enrolled cases with coordinators.

Treatment documentation team

• obtain information about inpatient from medical record and outpatient treatment from caregivers from days 1-7.

• fill the required case report forms.

Independent outcome assessment team

• assess all enrolled infants on day 2, 4, 8 and 15.

• communicate with hospital physician/nurse or study coordinator to confirm findings.

• fill the required case report forms.

• in case of serious adverse event, fill the required case report form.

• for critically ill infants, obtain the survival status after 14 days of initiation assessment and complete the required case report form.

Data management team

• check data after completion by each team regularly.

• clean data and upload cleaned data on central data management site on regular basis.

**Figure 1:**
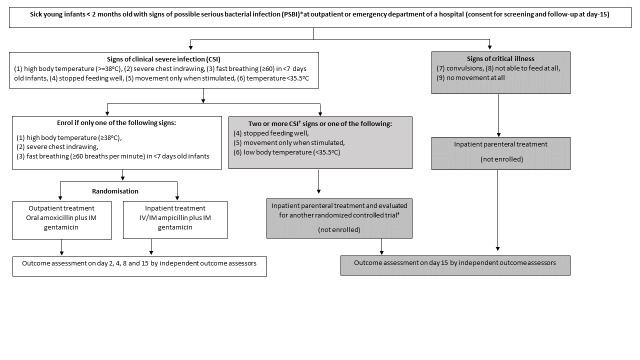
Study approach for screening, enrolment and management of sick infants. *Signs of Possible Serious Bacterial Infection (PSBI): convulsions, not able to feed at all, stopped feeding well, severe chest indrawing, body temperature ≥38°C, body temperature <35.5°C, movement only when stimulated, no movement at all, fast breathing in <7 days. **^†^**International Standard Randomised Controlled Trial Number (ISRCTN) Registry [29]. CSI – clinical severe infection, IV – intravenous, IM – intramuscular

See the study approach for screening, enrolment and randomisation in **Figure 1** and the implementation strategy in **Table 2**. In addition, study information in terms of objectives and procedures will be posted in the labour and delivery wards, antenatal care clinics and postnatal clinics in the participating hospitals.

**Table 2:** Implementation Strategy for Intervention and Control Arms

Activity	What	Who	When	Where	How
Case identification	Detection of cases	Screening, Enrolment and Randomisation Team with hospital staff	The sick young infant is brought by the family for consultation	OPD or general or paediatric emergency department of the hospital	Clinical assessment
Screening, randomisation and enrolment	Detection of eligible cases	Screening, Enrolment and Randomisation team	The young infant is seen by the hospital staff and the study staff	OPD or general or paediatric emergency department of the hospital	Clinical assessment, administering informed consent, randomisation and enrolment
Intervention arm (outpatient): treatment provision	Injection gentamicin and oral amoxicillin	Hospital / study staff and mothers	After enrolment and consent	First doses and subsequent injectable doses are given at the place of enrolment and the remaining oral doses are given at home by the mother / caregiver	Practical demonstration of giving the first dose in front of the mother / caregiver
Control arm (inpatient): treatment provision	Injection gentamicin and injection ampicillin	Hospital / study staff	After enrolment and consent	Hospital	Giving injectable therapy
Treatment documentation	Follow-up for treatment documentation	Treatment Documentation Team	2, 4, 8 post-randomisation	Intervention arm: patients' homes and OPD	Intervention arm: asking mothers and checking medicine bottles
				Control arm: hospital where the patient is admitted	Control arm: hospital inpatient treatment records
Supervision	Sub-sample on follow-up days for quality assurance	Study supervisor	2, 4, 8 and 15 post-randomisation	Intervention arm: patients’ homes and OPD	Verification and substantiating assessment
				Control arm: hospital where the patient is admitted and the patients’ home after discharge	
Outcome assessment	Outcome assessment	Independent Outcome Assessment Team	Day 2, 4, 8, 15 post-randomisation and if the patient deteriorated in between	Intervention arm: patients’ homes or a hospital if the patient was admitted	Clinical assessment and filling outcome assessment form
				Control arm: hospital where the patient is admitted and at home after discharge	

*OPD – outpatient department

### Randomisation to treatment arms

Participants will be individually allocated to inpatient or outpatient treatment with a block randomisation scheme. WHO staff in Geneva not associated with site work prepared the randomisation list in blocks of 2, 4, 6 and 8 and shared it with the Regional Triangle Institute (RTI), the study Data Coordination Centre (DCC). Allocation concealment was ensured using QR randomisation codes. Randomisation was in a 1:1 ratio. RTI produced a list of encrypted QR codes and has these printed on labels based on the randomisation scheme for each site and study. RTI transmitted the printed QR code with blue colour labels to the sites. There will be three labels per infant: one each for the log register, patient card, and hospital file or outpatient department (OPD) slip.

Each hospital within the site will have a single set of QR codes that all screening and enrolment team members will use, even if the screening and enrolment are taking place in different locations within the hospital. After determining eligibility, the data collector will take the QR code label next in the numeric sequence from the list at their facility and scan this to obtain the randomisation number.

### Sample size

The sample size was calculated to test both the superiority and noninferiority hypotheses. The target enrolment is 7000 young infants (3500 in each study arm) with low-mortality risk signs. This sample size will allow us to meet the superiority and inferiority targets given in **Table 3**.

**Table 3:** Sample size calculation for the trial design

Trial design	% poor clinical outcome in the control arm [6,7]	% poor clinical outcome in the interventional arm	Power	% lower outcome or noninferiority margin	Sample size needed
Superiority	6.0	4.0	90%	30	6270
Superiority	6.0	4.5	80%	25	6938
Noninferiority	6.0	6.0	90%	1.8	5966
Noninferiority	6.0	6.0	80%	1.5	6202

### Dosage of medicines to be used

Infants randomised to the intervention arm will receive the first dose of antibiotics in the outpatient department. Those randomised to the control arm will be hospitalised and receive injectable antibiotics (**Table 4**).

**Table 4:** Treatment arms

	Hospital arm		Outpatient arm	
	Injection Gentamicin	Injection Ampicillin	Injection Gentamicin	Oral Amoxicillin
Route	IM	IM / IV	IM	Oral
Frequency (per day)	Once	Two to Four*	Once	Twice
Dose by weight	Strength, 40 mg / ml	Strength 250 mg / 1.5 ml	Strength, 40 mg / ml	250 mg Dispersible tablet
1.5-2.4	0.2	0.8	0.2	1 / 2 tablet
2.5-3.9	0.4	1.2	0.4	1 / 2 tablet
4.0-5.9	0.6	1.5	0.6	1 tablet

IM – intramuscular, IV – intravenous, mg – milligramme, ml – millilitre

*Depending on the age of the child, for the hospitalised child, twice daily in the first week of life, thrice daily in 2-4 weeks of life, four times daily in 4+ weeks of life [4].

### Treatment documentation

The study treatment documentation and compliance team will document the treatment received in the hospital (control) and home (intervention) from days one to seven after enrolment. For infants in the control arm, the documentation of treatment received will be captured from the hospital inpatient treatment charts. For infants in the intervention arm, the documentation of treatment received will be captured in the OPD physician notes or reported by the mother.

### Study outcomes

The poor clinical outcome will be defined as: 1) death at any time from randomisation up to day 15 of initiation of therapy, or any sign of critical illness (no movement at all, unable to feed at all, or convulsions) on day 2, 4 or day 8 post-randomisation, or 2) any sign suggestive of another serious infection, e.g., meningitis, bone or joint infection on day 2, 4 or day 8 post-randomisation, or 3) any new CSI sign on day 4 or day 8 post-randomisation, or 4) persistence of the presenting CSI sign on day 8 post-randomisation.

### Outcome assessment

The proposed trial cannot be blinded due to the nature of the treatment. It is, therefore, important to incorporate measures to reduce potential measurement bias in the design of the study. We will use independent outcome assessors (IOA), who will be trained nurses or physicians or clinical field workers and will be independent of the enrolment and treatment to evaluate study outcomes. IOAs will be trained in standard management and study procedures. Outcome assessment will be carried out by independent outcome assessors (IOAs) and members of the outcome assessment team, who will visit all enrolled young infants on days 2, 4, 8 and 15 after enrolment. In the hospital arm, the outcome visits on days 2 and 4 will be done in the hospital, while the days 8 and 15 visits will be done at home (or in the hospital or where ever the child may have moved to). In the outpatient arm, the outcome visit on day 2 will be done when the infant returns to the hospital to receive the day 2 gentamicin injection. The outcome visits on days 4, 8, and 15 will be done at home or where ever the infant may have moved to.

The IOAs will only ascertain outcomes per the criteria mentioned above under outcomes. A case will be considered a poor clinical outcome if the IOA confirms it.

### Follow-up

All infants will be followed up till day 15 after initiation of therapy. Infants in the intervention (outpatient) arm will be asked to return to the hospital for treatment and assessment by the hospital physician / nurse on days 2 and 4 of treatment initiation. Infants will be assessed for the presence of any adverse event during scheduled follow-up visits to the hospital OPD, hospital visits by the family for care-seeking at any time during the follow-up period, or reported to the IOA team during a home visit and referred for care. These will be managed by the treating physician as per routine practice and followed up till resolution.

Infants in the inpatient arm will be followed up and managed as per routine hospital procedures.

All efforts will be made to follow all the enrolled young infants in the study. In case a young infant does not come to the hospital / health facility on the day of follow-up, the study team will contact them by phone and counsel parents/caregivers to bring the infant to the hospital / health facility. Transportation or transportation costs will be provided to families so that it is not a burden on the family to come for follow-up.

### Data handling and confidentiality

Data will be collected prospectively using electronic devices. Protecting the confidentiality of the data collected in the study will be a high priority. The consent form and any other forms linking participant personal information to study ID numbers will be kept in securely locked filing cabinets. Regular backups of the existing data will be done at appropriate intervals. All computers being used in the study will be password protected and will have restricted access to specific study staff to protect confidentiality. Participants’ names or identifiers will not be used in any publication. The paper-based log books will be kept for 10 years from the date of screening to complete all analyses, and then these will be destroyed.

### Data management

All study data will be collected on electronic case report form (CRF). Each site will collect data using Android-enabled devices using the PSBI data management system developed on the Tangerine software platform (http://www.tangerinecentral.org/ and https://docs.tangerinecentral.org/). Quality control will be performed on-site: each research team member will be responsible for data quality checks and ensuring that all forms are correctly filled out. A central data repository will be created. All efforts will be made to minimise missing data. There will be no imputation of missing data. RTI will develop and support a common data management system in collaboration with WHO and site investigators, conduct statistical analysis and support data monitoring, and perform on-site monitoring visits to improve the quality of data. Standardised data collection tools will facilitate the study data to be entered directly into the electronic forms using laptops, notebooks, or other portable electronic equipment. While centralised, however, each site will also own and support the data collection system. The data management system will be maintained and managed locally at each site, with support, as needed, from RTI technical staff. RTI will develop a data monitoring plan in collaboration with the WHO and study investigators to provide regular reporting on data recruitment, adherence to the study protocol and other metrics deemed important. RTI will provide periodic additional reports as needed (i.e., in preparation for the DSMB or Steering Committee meetings) from the centralised monitoring.

### Data analysis

Means and proportions of baseline characteristics by inpatient and outpatient groups will be presented. The primary analyses will be intention-to-treat to compare the risk of poor clinical outcomes between the intervention and control arms. Risk difference and the 95% confidence intervals will be presented to assess. Those who will be lost to follow-up and from whom consent would be withdrawn will not be included in the per-protocol analysis. The treatment outcomes between control and intervention groups will be compared, and the difference in the risk of poor clinical outcomes together with 95% confidence intervals, will be calculated. Secondary analyses will be performed to investigate the effect on secondary outcomes (predictors of poor outcomes); that is, univariate and multivariate regression analyses will be performed to identify predictors of poor clinical outcomes.

### Quality control and assurance

All study teams (screening, enrolment, and outcome assessment teams) will have study supervisors who will support adherence to the manual of operations. The quality assurance team will conduct regular standardisation exercises, oversight and monitoring of all study activities through regular and random visits and checks of proportion (10%) of all completed study forms. A site preparation review will be conducted before the initiation of the study. This will include the standardisation of practices and measurements. WHO staff and study consultants will provide external oversight and support to ensure the quality of study implementation.

### Quality of care in hospitals

The hospital infant care services will be strengthened regarding human resources and capacity building through training, processes and standard operating procedures (SOPs), ensuring continuous availability of standard quality antibiotics and basic support for routine care. The “minimum” quality of care will be according to the WHO pocketbook for hospital care for children [4], which includes keeping the baby warm and providing kangaroo mother care to prevent hypothermia (in those who need it), encouraging a mother to breastfeed frequently to prevent hypoglycaemia, fluid management when required, basic laboratory support, and oxygen therapy when needed [4]. The hospital team will be oriented on the study protocol and recommended treatment protocols, including information on indications for changing the treatment regime. Regular visits to assess the quality of care at the hospitals will be made by an experienced paediatrician / neonatologist. Medical equipment provided to the participating hospitals for the study will remain there after the completion of the study.

### Training of health workers and study staff

The study staff who will perform screening, enrolment, and outcome measurement will be trained using standardised study SOPs. Data will be collected by research staff on standardised data collection forms. During training, emphasis will be placed on maintaining Good Clinical Practice (GCP) standards. All research staff will be trained in rapport-building and communication with mothers / caregivers. The screening and enrolment study staff will be trained in introducing the study to potential participants, administering the consent form, assessing for clinical signs and eligibility, and completing all relevant study forms. The outcome measurement staff will be trained in the definitions of outcomes and on their standardised assessment, as well as completion of the outcome assessment forms.

### Standardisation and refresher training

Periodic standardisation exercises every quarter will be carried out for study staff, supervisors, and IOAs to identify clinical signs to maintain their clinical skills. The study sites will also develop a system of periodic refresher training for health workers, supervisors and IOAs through clinical practice and / or video demonstrations. In addition, periodic centralised standardisation exercises and refresher training will also be conducted by WHO for the study staff.

### Logistics and commodities

All sites will be equipped with necessary medicines, equipment and logistics for pneumonia case management, such as ampicillin and gentamicin injections, oral amoxicillin 250 (or 125) mg dispersible tablet, pulse oximeter with accessories, digital and mercury thermometers, digital weighing scales, respiratory rate counting timers / digital timers and other necessary materials centrally through the WHO supply division to maintain standardisation. The study teams will monitor the equipment for periodic standardisation and replacement of faulty devices, if any. The consumables will be replenished as per need.

### Supervision

All study staff will be supervised by a study supervisor. All young infants who are assessed as potential enrolment will be periodically validated by a supervisor to ensure quality. Supervised accompanied visits and independent unaccompanied visits will be carried out regularly by the supervisors. Video calls or video clips of signs, eg, chest indrawing, will also be used in real-time to allow supervisors to monitor the correct identification of signs from time to time. Principal investigators and co-investigators will also make random visits to check the project's performance and quality.

Periodic meetings of the research implementation teams will be held to review enrolment, performance, observations and follow-up problems and identify solutions / required actions to overcome those. Action points will be followed up on in the next meeting.

### Monitoring

Monthly progress reports will be submitted by all the sites to the coordinating site at the WHO, which will be reviewed, and feedback will be provided. Regular conference calls will be held with all sites to discuss the progress and critical issues of the study. Databased monitoring, as well as verification of serious adverse event (SAE) forms, will be carried out by the DCC in collaboration with the WHO study coordination team. Investigators and study coordinators will randomly visit the field sites to observe study staff, supervisors and IOAs performing their activities. They will cross-check a small proportion of collected CRFs and share observations with supervisors and IOAs.

### External monitoring

The WHO study coordinating team will visit each site before the initiation of the study to assist in the preparation and planning of the site implementation of the protocol and study procedures. Due to restrictions on travel because of the COVID-19 pandemic, virtual visits and the monthly online meeting will be held. Country-based or other external WHO monitors will visit each site at least once or twice a year to monitor the progress and observe the procedures using a checklist to monitor clinical trials. It will include assessment and enrolment procedures, clinical practices such as counting respiratory rate, identifying danger signs, conducting pulse oximetry, and review other study procedures, and data management. Various logbooks will be checked. A proportion of completed electronic CRFs will be checked, and all SAE reporting forms will be reviewed. WHO monitors will also observe physician awareness and will meet with the regular hospital staff. In addition, they will make a home visit to those who are receiving treatment in the intervention group and talk with the parents. When possible, the monitors will meet with the community groups’ members, ministry and district health officers, and programme managers at different levels. Recommendations arising from the site monitoring visits will be shared and discussed with the principal investigators and other project staff at the site office, and follow-up actions will be undertaken.

### Ethical considerations

The protocol has been approved by all the local institutional ethical review committees at the study sites and the WHO ethical review committee. Where required, national and regional / state approvals have been obtained. The safety of enrolled infants in this trial will be ensured by close monitoring and follow-up. The trial will follow CIOMS and Good Clinical Practice guidelines.

### Informed consent

Informed written consent will be obtained from parents / caregivers by a study nurse / physician at two stages, first at the time of screening and later at the time of enrolment. Detailed verbal communication in the caregivers’ native language will be provided to ensure comprehension of the trial and study procedures. If the participant is illiterate, then a witness signature will be required. All consent forms will be translated into local languages. Parents / caregivers of sick young infants will be provided basic information about the study and invited to consent for the screening. Parents/caregivers of infants found to be eligible after the screening will be provided full information about the study and invited to have their infant participate in the study. The eligible infants will be enrolled if their parents / caregivers provide informed consent and will be randomised to either one of the treatment arms. Illiterate parents / caregivers will be asked to give a thumbprint on the consent form; literate parents/caregivers will be requested to sign the consent form. Study staff will be trained in the study methodology, including obtaining consent.

### Patient safety

The study procedures and data collection will not pose any significant physical, psychological, social, legal or other kinds of risks to the participants. Clinical assessment and illness management, including the provision of drugs to be used in this study, are routinely used in clinical practice. Every enrolled subject will be followed up till the completion of treatment and assessed on day 15 of the enrolment. Treatment failures will receive an appropriate change of antibiotic therapy according to standard clinical practice, and those who need hospitalisation will be referred and admitted to the sub-district / district / tertiary care hospitals immediately for appropriate management, as required. Other discomforts to caregivers might include a longer waiting time for data collection. However, the procedures will be explained in detail, and any queries by parents will be answered. The mobile phone number of the responsible study staff and study supervisors will be provided to all participants’ caregivers so that they could reach them if needed.

We will counsel and empower the mother / caregivers / families at enrolment and on all follow-up visits to 1) recognise danger signs or signs of illness and seek care and 2) know when to return to the hospital for follow-up care. We will train the mother / caregiver / family on the quantity, frequency and process of giving oral antibiotics at home. Systems will be in place in the hospital to provide emergency and rescue care in case of any adverse event or facilitate referral to a higher facility if required.

### Adverse events – documentation, reporting and response

Any adverse event that occurs after enrolment will be recorded on an adverse event reporting form by the treating nurse / physician. In case of an SAE, the study staff will contact the study supervisor and IOA. The IOA will document the SAE and convey the information to the study coordinator / investigator. An SAE, like death, anaphylactic reaction, severe diarrhoea, and a disseminated or severe rash, will be reported to WHO within 48 hours of the occurrence. These cases will be (except the ones who unfortunately die) referred for appropriate treatment and will be followed up. In case of other minor adverse effects, such as mild rash etc., the treatment will be continued. WHO will report SAEs to the Data Safety Monitoring Board (DSMB).

### Withdrawal from the study

Parents / caregivers can withdraw at any time after enrolment, and those who withdraw at any stage from the study will continue to receive free-of-charge standard treatment and treating physicians will follow them as per hospital standard procedures. These cases will be excluded from the per-protocol analysis but will be included in the intention-to-treat analysis.

### Non-enrolled cases

All critically ill cases will be followed up by day 15 after the initial screening to obtain information about their survival status. Consent will be obtained from them about collecting this information. The survival status of each non-enrolled infant will be collected through a home / hospital visit (wherever the infant will be present) or by calling his / her parents / caregivers. This information will be used for the secondary analyses.

### Steering Committee

This will comprise all principal investigators from study sites, consultants, donor representatives and WHO technical staff (secretariat). WHO will be responsible for organising Steering Committee meetings before study implementation, 9-12 months into the study and at the end of the study or as required during the study. This committee will be responsible for designing and implementing the study harmoniously. Study principal investigators will be responsible for contributing to the development of the research proposal, study manual, data management system, implementation of the intervention, outcome measurement and data collection, data analysis and interpretation and dissemination of results. All activities will be facilitated and supported by WHO. Every month, the sites will submit a brief status report to WHO. A formal progress report will be submitted by each site every year.

### Technical Advisory Group (TAG)

This group will be set up that will include three external experts in the field. The TAG members will serve in their capacity and will review the final research protocol for any major concerns before trial implementation. TAG members’ terms of reference also include review of the manual of operations, CRFs and consent forms and advice on practical issues in implementing the trial in the field. WHO will serve as secretariat to this group and organise two meetings, one before the study starts and one after one year of study or as required during the study.

### Data safety monitoring board

An independent DSMB will be constituted to monitor the trial at regular intervals.

### Community and health sector engagement and dissemination of results

In the clinical settings where this study will be conducted, before its initiation, the local investigators will engage in dialogue with hospital health staff, ministry of health staff, community representatives, community-based organisations and non-governmental organisations working in the area to explain to them various aspects of this study.

Public health administrators will be part of the research teams and health care staff. The health facility administrators and professional health facilities linked with the study will also be sensitised about this research through personal contacts and sensitisation meetings. They will be informed that some patients with PSBI would be referred to tertiary care hospitals, and assistance with management will be needed for those children.

The potential audience for dissemination will be government officials, policymakers, academics, researchers, the local community and other voluntary organisations involved with community-based services. To reach this varied audience, a multipronged dissemination strategy will be required. We will invite the audience to dissemination seminars, which will be organised at the end of the project. We will hold meetings with the community members and their leaders for local communities. We will publish the findings of these studies in peer-reviewed journals, with local researchers as the lead authors. We will submit abstracts at national and international conferences.

### Trial registration

The trial is registered at the International Standard Randomised Controlled Trial Number (ISRCTN) registry as ISRCTN44033252.

## DISCUSSION

Neonatal sepsis and PSBI still cause a large number of deaths. However, not all infants with these conditions have access to health care services and treatment. More equitable access to diagnosis, standardised care and appropriate treatment are critical to reducing PSBI mortality. The following discussion outlines the efforts taken to safely and efficiently conduct this trial to rigorously assess the benefits of outpatient treatment of PSBI. The study's primary question is whether poor clinical outcome rates of low-mortality risk signs with outpatient treatment with simpler antibiotics are superior or at least non-inferior to the poor clinical outcome rates of PSBI treatment with the standard strategy of inpatient therapy in a hospital. We will compare the poor clinical outcome rates of PSBI in young infants with low-mortality signs on an outpatient basis with an injectable plus oral antibiotic with current management of hospitalisation and treatment with two injectable antibiotics. The study has been carefully developed and pragmatically designed with inclusion and exclusion criteria to allow as generalisable results as possible without putting young infants with PSBI at risk. All enrolled young infants in the intervention arm will be diagnosed with PSBI based on the WHO PSBI guideline [3,5]. The young infants in the intervention and control arm will be treated based on the WHO PSBI guideline [3,5] and the WHO pocketbook protocol [4], respectively.

Effective strategies ensure that infants receive timely diagnosis and appropriate treatment for PSBI even when a referral to a hospital is not available. Improving access to services and increasing awareness and demand for care within communities is crucial for the management of PSBI and for saving lives. In the absence of any of these, infants may never receive potentially lifesaving treatment, or it may be delayed until the disease becomes more severe. In a young infant, sepsis can progress quickly. This study will help identify those infants with PSBI who can be promptly diagnosed and treated at a health facility on an outpatient basis. It will reduce the unnecessary referral to a hospital, which families in low-resource settings find challenging to accept due to various reasons [19,30-33].

Our study has several strengths. First, it is a multicentre study to be conducted in six countries across Africa and South Asia with a fairly large number of patients. Therefore, results are expected to be generalisable across a wide range of low-resource setting contexts. Second, standardised training, supervision, oversight, and monitoring will be undertaken to ensure quality, consistency, harmonised trial procedures and implementation. Trained study staff will document treatment and follow-up enrolled cases in the trial. Outcome assessment by an independent assessor will minimise ascertainment bias. Supervisors will ensure that the protocol and standard operating procedures are followed, data are accurately collected, and the highest level of safety is provided. Third, in addition to regular site monitoring visits by study staff and investigators, the WHO monitoring and coordinating team in Geneva will be engaged to assist with close external monitoring of the study, including regular site monitoring visits to assess compliance with human subjects, other research regulations and guidelines, adherence to the study protocol and procedures, quality and accuracy of data collected, and quality of care and child safety. Fourth, strong collaboration across sites with monthly steering committee meetings with all investigators for early identification and resolution of issues and challenges will be undertaken. Finally, the study is being conducted in partnership with the local governments to ensure early buy-in and translation into policy.

There are also a few limitations. First, no laboratory or radiological assistance will be available to assist with the diagnosis. While microbiological and / or other diagnostic aids may add improved specificity to the clinical diagnosis of PSBI, peripheral health centres in low-resource settings do not have access to these investigations, and young infants are typically diagnosed based on clinical criteria alone. Second, the blinding of intervention therapy is not possible. Third, there may be an element of subjectivity involved in the assessment of poor clinical outcomes based on the presence or absence of clinical signs. To reduce bias, independent assessors will conduct outcome assessments in both intervention and control groups in a standardised manner. Finally, a potential limitation could be strengthening hospital-based care in the study facilities from an ethical standpoint, which may differ from other facilities that don’t get such support, making the findings less generalisable.

## CONCLUSIONS

If the results show that young infants with low-mortality risk PSBI signs can be effectively and safely treated on an outpatient basis, it may substantially increase access to treatment for families with poor access to health facilities. It may also reduce the human, financial and material costs to the health system and allow currently overloaded health facilities to focus on more critically ill infants. This evidence will contribute toward making a case for reviewing the WHO PSBI guideline [5].
